# Temporal Artery Ultrasound for the Diagnosis of Giant Cell Arteritis in the Emergency Department

**DOI:** 10.7759/cureus.42350

**Published:** 2023-07-24

**Authors:** Patricia Hernández, Nour Al Jalbout, Mark Matza, Minna J Kohler, Hamid Shokoohi

**Affiliations:** 1 Emergency Medicine, Massachusetts General Hospital, Harvard Medical School, Boston, USA; 2 Rheumatology, Massachusetts General Hospital, Harvard Medical School, Boston, USA

**Keywords:** temporal arteritis, giant cell arteritis (gca), temporal artery ultrasound (taus), emergency department (ed), halo sign, point-of-care ultrasound (pocus)

## Abstract

Giant cell arteritis (GCA), known as temporal arteritis, is a serious condition requiring immediate treatment to prevent complications. GCA can be difficult to diagnose, especially in emergency department (ED) settings where ophthalmology and rheumatology services may be unavailable. Temporal artery ultrasound (TAUS) is a valuable tool for diagnosing GCA. In the ED, TAUS can be used to quickly rule out GCA and avoid unindicated steroid treatment, which can cause serious morbidity in elderly patients. This article discusses the use of TAUS for evaluating patients with suspected GCA in the ED and its potential to expedite treatment and ensure appropriate, timely follow-up for patients with this potential vision and life-threatening condition.

## Introduction and background

Giant cell arteritis (GCA), previously known as temporal arteritis, is a serious autoimmune vasculitis that affects medium and large-sized blood vessels, particularly the external carotid artery branches [1,2]. Presenting symptoms may include new onset headache, scalp tenderness, vision changes, jaw claudication, and occasionally systemic symptoms such as fever, fatigue, and weight loss [3,4]. Ocular complaints include vision loss of varying degrees, from amaurosis fugax to complete blindness, and can sometimes include diplopia and eye pain [4]. If left untreated, GCA can lead to vision loss secondary to arteritic ischemic optic neuropathy in 15-25% of cases [3,5]. Other serious complications, such as stroke, can also occur due to carotid and vertebrobasilar vasculitis [6]. GCA is primarily seen in patients over 50, with a higher incidence in women and those with a history of polymyalgia rheumatica [7,8]. The lifetime risk of developing GCA is approximately 1% in women and 0.5% in men [6,9,10].

In the emergency department (ED), the timely recognition and treatment of GCA is crucial as it is a potentially sight-threatening condition. Non-invasive vascular imaging, including temporal artery ultrasound (TAUS), is increasingly used to support the clinical diagnosis of GCA in outpatient clinics. TAUS is non-invasive, widely available, cost-effective, and easy to perform, with a high sensitivity and specificity for diagnosing GCA [11]. According to a descriptive review examining patients in rheumatology clinics, TAUS has a diagnostic sensitivity of 89% and a specificity of 94% for the GCA [12,13]. The European Alliance of Associations for Rheumatology (EULAR) recommends using TAUS as a first-line investigation for suspected GCA, and the American College of Rheumatology (ACR)/EULAR Classification Criteria for GCA now includes the halo sign on TAUS as a diagnostic criterion [3]. The halo sign refers to a hypoechoic vessel wall thickening of the intima-media complex (IMC), representing the granulomatous inflammation characteristic of the GCA [14].

In this article, we will review the use of TAUS in patients with suspected GCA and its role as a practical screening test for GCA in the ED. By understanding the diagnostic principles using TAUS in the ED, clinicians can assist in the timely diagnosis of GCA, mitigating the risk of irreversible vision loss.

## Review

Imaging diagnosis of GCA

Traditionally, the gold standard for diagnosing GCA was a temporal artery biopsy (TAB), which involves taking a small sample of the temporal artery and evaluating it for typical histological findings [4]. However, advances in imaging technologies have allowed for non-invasive diagnosis of GCA using techniques such as computed tomography (CT), magnetic resonance imaging (MRI), and 18F-fluorodeoxyglucose (FDG) positron emission tomography (PET) [12]. CT angiography has a pooled sensitivity of 95% and specificity of 100% and may be useful in identifying extra-cranial manifestations of GCA such as aortitis or large-vessel vasculitis; however, its use is limited due to ionizing radiation [12]. MRI is useful in visualizing vessel wall thickening, and edema, assessing multiple cranial arteries simultaneously, and in long-term monitoring of GCA and has a pooled sensitivity of 73% and specificity of 88%; however, access to MRI and expertise in interpreting MRI images may vary upon institution [12,14]. MRI use is also limited in patients with renal failure, implanted devices, or claustrophobia [12]. While these imaging modalities can potentially improve the diagnosis of GCA, their use is limited due to high cost and low availability [14]. Therefore, the ACR and EULAR have set criteria for diagnosing GCA that incorporate clinical, laboratory findings, and imaging results and suggested TAUS as first-line imaging for GCA. The ACR/EULAR Classification Criteria for GCA most recently included the halo sign on TAUS as a diagnostic criterion [3]. It is important to note that while a TAB is no longer considered the gold standard for diagnosing GCA, it may still be necessary to confirm the diagnosis or rule out other conditions.

Ultrasound diagnosis of GCA

TAUS was first used for diagnosing GCA in 1997 [15]. With advances in imaging resolution and greater availability of TAUS, it can become the primary imaging modality for patients suspected of having GCA. In an observational study of 451 patients seen in the rheumatology clinic setting, Aranda-Valera et al. found that TAUS had a sensitivity of 91.6% and a specificity of 95.8% for diagnosing GCA [13]. Based on a pooled analysis of patients with suspected GCA from 12 studies (N=965), Coath et al demonstrated moderate agreement of color doppler ultrasonography with temporal artery biopsy (κ = 0.44, 95% CI 0.38-0.50). They also demonstrated good interobserver and intra-observer reliabilities in live TAUS scanning exercises and excellent reliabilities when assessing acquired images and videos [16].

TAUS has several advantages over TAB in evaluating patients with suspected GCA. TAUS is non-invasive, inexpensive, and produces results quickly. At the same time, TAB can be less sensitive as it samples only a small segment of the artery and may result in false negatives due to skip lesions [17]. Furthermore, ultrasound provides real-time imaging and dynamic assessment of blood flow in the temporal artery. This real-time visualization allows for a more comprehensive evaluation of arterial abnormalities, aiding in the accurate diagnosis of GCA [18]. However, the accuracy of TAUS in diagnosing GCA can be affected by the skill and experience of the clinical sonographer performing the scan. Emergency medicine physicians (EPs) can become proficient in performing and interpreting TAUS in the ED to improve diagnostic reliability and clinical utility. While previous research has examined the use of TAUS in diagnosing GCA in rheumatology clinics, no studies to date have characterized its role in the ED setting. However, a recent case report of GCA diagnosed on bedside ultrasound in the ED suggests that TAUS can be an effective tool utilized by EPs to identify this emergent diagnosis [19].

Ultrasound scanning protocol

**Figure 1:**
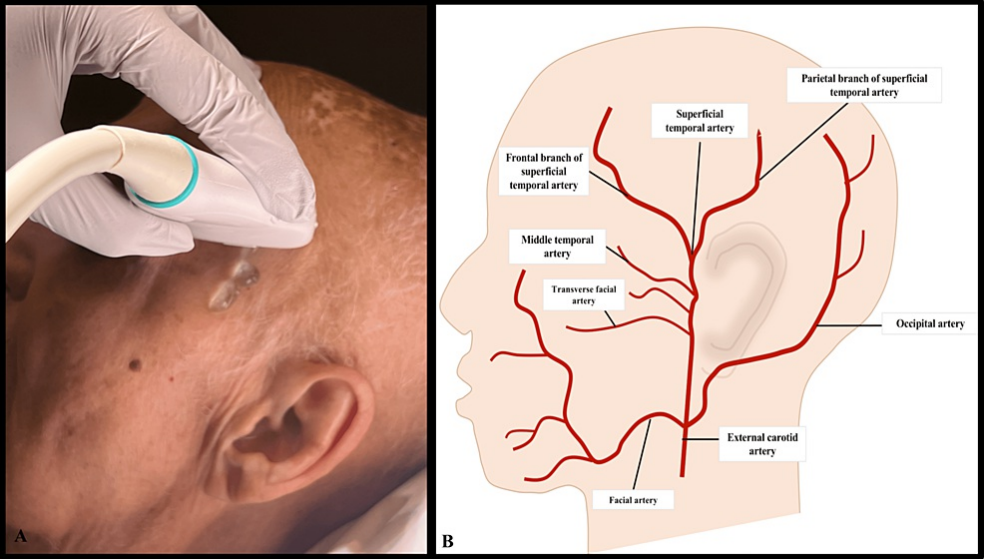
GCA scanning protocol: (A) Depiction of linear probe placement for GCA scanning protocol on patient; (B) Anatomy of temporal artery Image credit: (A) Authors; photo consent obtained from patient; (B) Original sketch by authors GCA: giant cell arteritis

The TAUS protocol for diagnosing GCA in the ED involves using a high-frequency linear transducer to scan both superficial temporal arteries. The patient is positioned supine or sitting, and the ultrasound probe is applied gently to the temporal artery with the color Doppler turned on. The probe should be used lightly to minimize pressure on the temporal artery. The common superficial temporal artery is anterior to the ear and branches into the frontal and parietal branches at the level of the ocular mid-commissure (refer to Figure 1a which depicts probe placement on a patient). The anatomy of the temporal artery and its branches is depicted in Figure 1b. As part of the protocol, all three temporal artery branches (common, superficial, parietal, and frontal) should be imaged in longitudinal and transverse views. The exam typically begins with the common superficial branch in either longitudinal or transverse view and extends superiorly to the distal parietal branch. The probe is then rotated to evaluate the parietal branch back to the branch point. The length of the frontal branch is then assessed, extending distally and then back proximally before returning to the common superficial branch (Figure 1). Some patients with GCA may experience focal tenderness in certain areas, which should be carefully examined during the ultrasound scan.

Ultrasound findings in GCA

**Figure 2:**
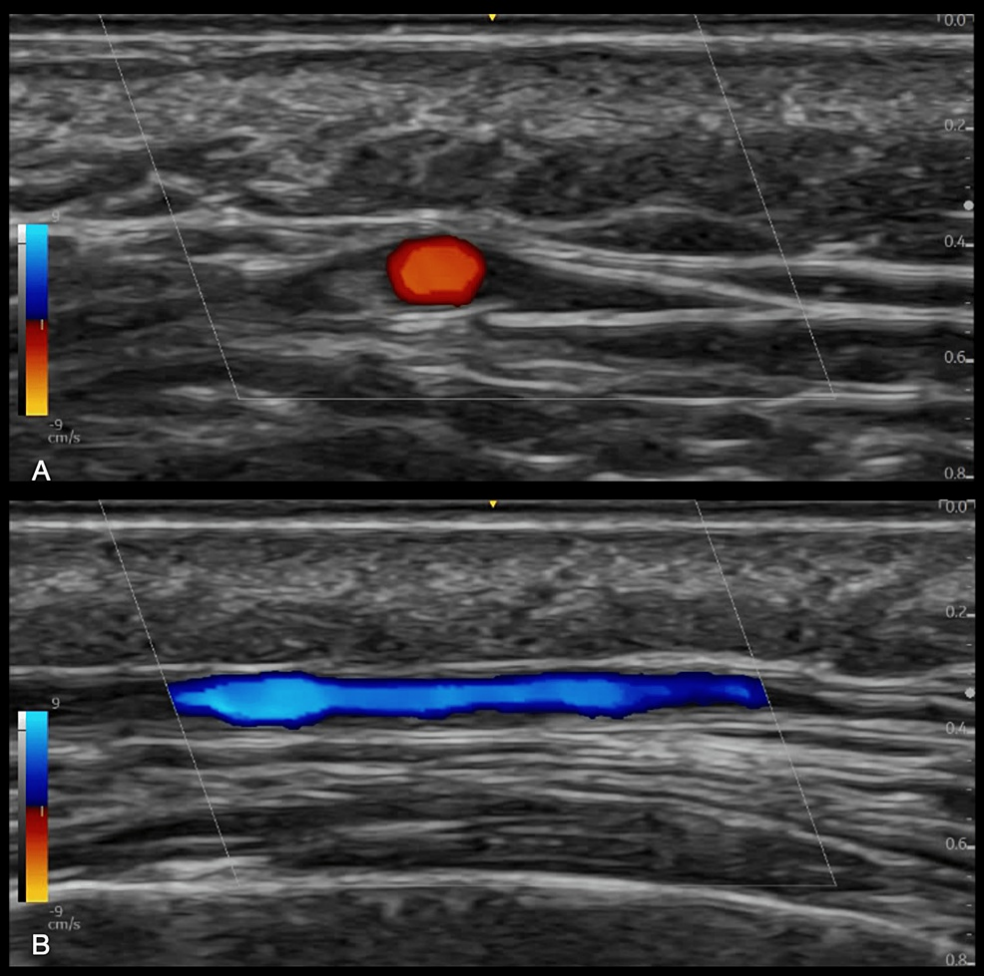
Normal superficial temporal artery. (A) Transverse view, (B) Longitudinal view Image credit: Authors; consent taken from patient.

The temporal artery is a superficial artery located around 4-5 mm deep in the skin, making it an easy target to identify in TAUS (refer to Figure 1b for the relative anatomy of the superficial temporal artery). The gray-scale image of a normal temporal artery displays a very thin-walled artery and anechoic lumen (Figure 2).

**Figure 3:**
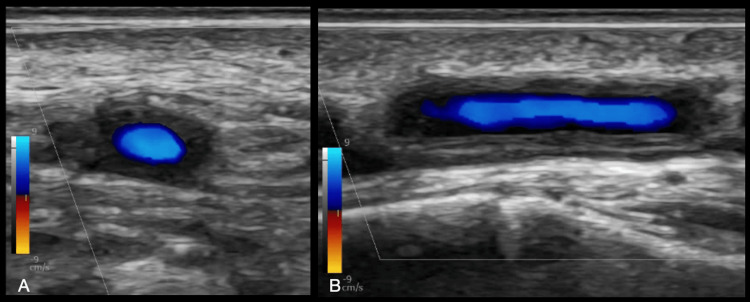
Halo sign in TAUS; hypoechoic, homogenous thickening of the superficial temporal artery. (A) transverse view, (B) Longitudinal view Image credit: Authors; consent taken from the patient with confirmed giant cell arteritis. TAUS: temporal artery ultrasound

**Figure 4:**
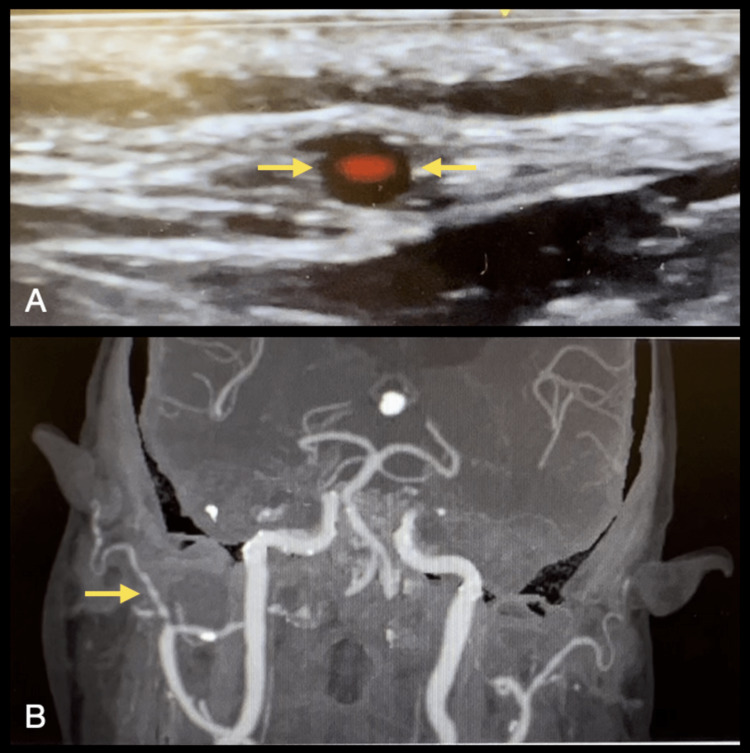
(A) Halo sign of the superficial temporal artery in an 89-year-old man with a history of polymyalgia rheumatica who presented to the ED with an acute painless right monocular vision loss and temporal tenderness. (B) Contrasted CTA revealed a beaded appearance of the right superficial temporal artery suggesting GCA. Image credit: Authors; consent taken from the patient with confirmed GCA. CTA: computed tomography angiography; GCA: giant cell arteritis

The presence of circumferential hypoechoic vessel wall thickening associated with the reduced luminal size suggests the GCA diagnosis [20]. The hypoechoic, homogeneous thickening of the vessel wall (intima-media complex (IMC)) is the “halo sign,” the most specific finding in TAUS (Figures 3A, 3B, 4A). A color Doppler scan helps visualize the arterial wall thickness and periarterial edema. Figure 4B depicts the beaded appearance of the right superficial temporal artery in a patient who presented to the ED with acute painless right monocular vision loss on contrasted CT angiography (CTA).

The halo thickness should be >0.3mm (varies by branch) [21]. A halo thickness of 0.7mm or more predicts a positive TAB result [21,22]. Ensuring that the gray-scale gain setting allows visualization of the intima medial wall caliber is imperative. If the gain is too low, this could cause a false halo sign.

**Figure 5:**
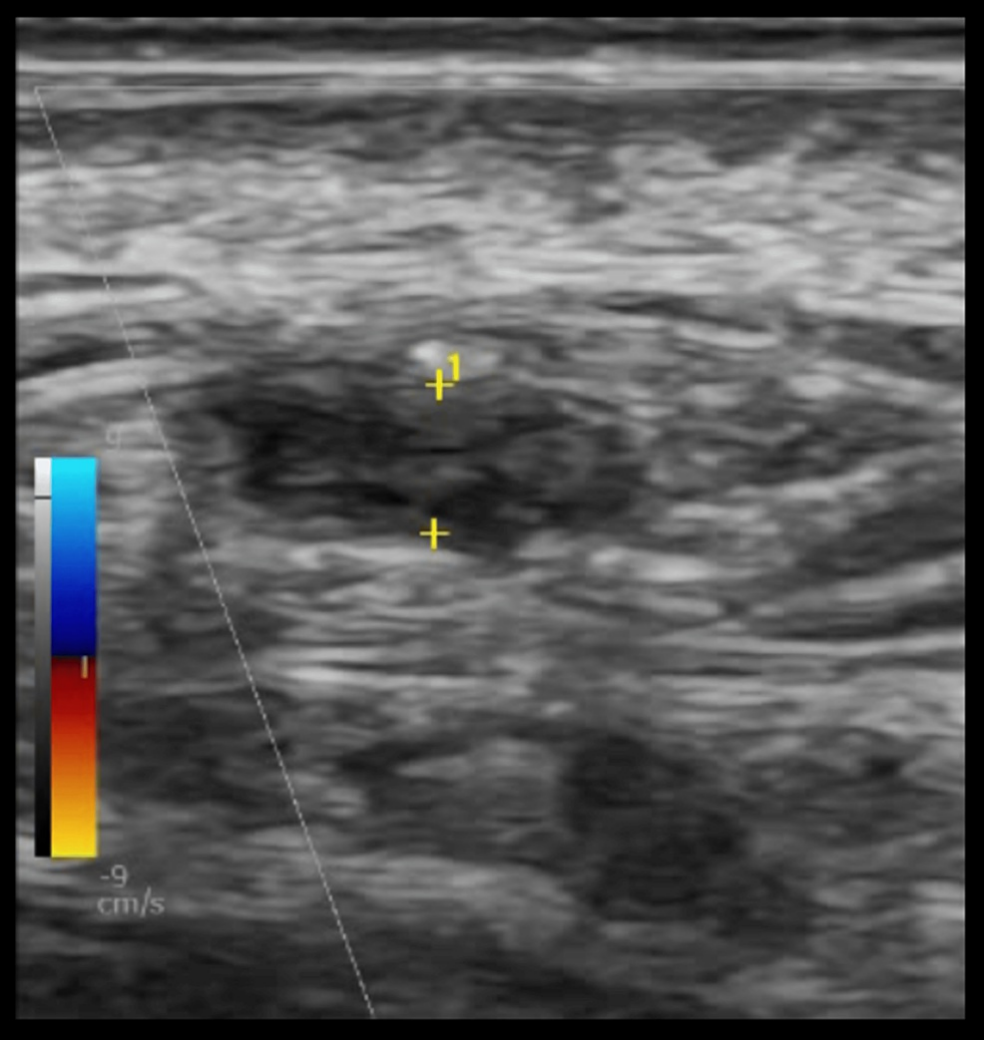
Compression sign of the superficial temporal artery in transverse view Image credit: Authors; consent taken from the patient with confirmed giant cell arteritis.

A normal artery will fully compress and disappear by applying pressure using the transducer. In contrast, in cases with GCA, the temporal artery wall may remain persistently visible when compressed with a transducer, a phenomenon known as the "compression sign" (Figure 5). This sign has a sensitivity of 75-79% and a specificity of 100% for diagnosing GCA.

If the halo thickness on TAUS is indeterminate and there is still a strong clinical suspicion for GCA, alternative modalities should be pursued to make the diagnosis. Additional imaging diagnostic criteria per the ACR/EULAR guidelines include Fludeoxyglucose F18 (FDG)-positron emission tomography (PET) activity throughout the aorta, and bilateral axillary involvement on imaging defined as stenosis, occlusion, or aneurysm on angiography (CT, MRI, or catheter-based) or ultrasound [23].

Discussion

GCA is a type of vasculitis that can cause serious complications, including vision loss, if not treated promptly. Several studies have examined diagnostic algorithms and fast-track pathways (FTPs) to improve morbidity and reduce costs in patients with GCA.

Using efficient diagnostic strategies, such as TAUS, has improved patient outcomes in GCA. In a study examining the impact of TAUS on the survival of patients with GCA, survival rates were notably higher at five and 10 years (79.5% at both cutoff points for patients diagnosed using the ACR criteria and TAUS within seven days of symptom onset compared to those diagnosed using the ACR 1990 criteria alone (53.4% and 36.7%, respectively) [24]. In the study, TAUS was performed by a rheumatologist with extensive ultrasound experience; however, these findings demonstrate the value of TAUS for the rapid diagnosis of GCA and the prevention of morbidity and mortality.

FTPs have also been shown to improve outcomes for GCA patients, including reduced time from symptom onset to diagnosis, reduced permanent vision loss, and reduced inpatient stay and costs. Two retrospective studies have demonstrated how, with the inauguration of outpatient FTP clinics and the utilization of TAUS as a screening tool for GCA, the rate of permanent vision loss fell dramatically from 37% to 9% and from 19% to 2% [5,25]. Matza et al. found that evaluating and managing patients with GCA involved a multidisciplinary collaborative approach among several practitioners, with emergency medicine providers playing an essential role in diagnosing and referring patients with suspected GCA [26]. This can mitigate a prompt diagnosis and timely treatment with a consult with rheumatology or ophthalmology. EPs play a vital role in the evaluation and management of GCA patients. They should be proficient in using TAUS and be familiar with the FTPs options in diagnosing and managing GCA.

GCA can be difficult to diagnose, especially in clinical settings where ophthalmology and rheumatology consultations are not readily available. In these cases, TAUS can be a valuable tool for diagnosing GCA. Ultrasound is readily available in the ED. It can be used to quickly rule out GCA and avoid unnecessary steroid treatment, which can cause severe morbidity in elderly patients, including infections, bone loss, and poor blood sugar control. EPs trained in ultrasound can adopt TAUS to identify patients with possible GCA by looking for halo and compression signs where time is of the essence. While GCA is rare, emergency medicine trainees can be taught a focused scanning protocol to assess GCA using TAUS. There needs to be more data on the frequency of use and diagnostic accuracy of ultrasound for GCA diagnosis by EPs, but with proper training, EPs can become proficient in TAUS and use it as a diagnostic tool for GCA. These findings highlight the vital roles of EPs in prompt diagnosis and timely treatment while consulting with rheumatology or ophthalmology and integrating TAUS and FTPs’ diagnostic strategies in evaluating and managing patients with suspected GCA.

## Conclusions

TAUS is a valuable non-invasive tool for assessing suspected GCA that streamlines management and consultations if positive in the presence of a suggestive clinical presentation. The use of TAUS in the ED potentially enhances patient care by narrowing the differential diagnosis and facilitating prompt diagnosis and timely management of GCA, ultimately improving patient outcomes. Furthermore, TAUS can help optimize resource utilization in the ED setting by potentially decreasing the length of stay, expediting discharges, and optimizing rheumatology and consultation pathways by referring patients only when indicated. Furthermore, by utilizing TAUS, EPs can swiftly and non-invasively diagnose or rule out GCA, especially in settings where specialized consultations may be limited. This approach may further avoid hospital admissions and unnecessary temporal artery biopsies, reduce the risk of complications associated with steroid treatment, and expedite appropriate treatment and disposition decisions for patients with potential vision and life-threatening conditions.

To enhance GCA diagnosis and management in the ED, it is crucial to explore the efficacy of ultrasound implementation in this setting. While current literature primarily focuses on TAUS in rheumatology, its potential in the ED is promising. However, operator experience and proficiency in performing TAUS may vary, posing a limitation. Future studies should determine optimal training and skill requirements for EPs to ensure accurate and reliable results when diagnosing or ruling out GCA in the ED, considering its rarity in this setting. EPs can improve their ability to identify possible GCA cases by adopting a focused scanning protocol and receiving training on halo and compression sign recognition.
